# Optimization of Ethanol Detection by Automatic Headspace Method for Cellulose Insulation Aging of Oil-immersed Transformers

**DOI:** 10.3390/polym12071567

**Published:** 2020-07-15

**Authors:** Hanbo Zheng, Chuansheng Zhang, Yiyi Zhang, Jiefeng Liu, Enze Zhang, Zhen Shi, Guangqi Shao, Kuikui Shi, Jing Guo, Chaohai Zhang

**Affiliations:** School of Electrical Engineering, Guangxi University, Nanning 530004, China; hanbozheng@163.com (H.Z.); zcsgxu@163.com (C.Z.); yiyizhang@gxu.edu.cn (Y.Z.); liujiefeng9999@163.com (J.L.); enze_zhang1@163.com (E.Z.); eee0619@sina.com (Z.S.); 15871466826@163.com (G.S.); KuiShi_gxu@163.com (K.S.); guojing6a@163.com (J.G.)

**Keywords:** cellulose insulation, aging, headspace sampling, gas chromatography

## Abstract

The method using ethanol to evaluate the cellulose insulation aging condition of oil-immersed transformers has been proposed. At present, the dominating method for detecting ethanol in insulating oil is to use headspace–gas-chromatography–mass-spectrometry (HS-GC-MS). However, the problem of quantitative inaccuracy will be sometimes encountered in the actual detection process due to improper instrument parameter setting and improper manual operation. In this study, as an aging marker, ethanol in transformer insulating oil was separated by using VF-624 ms capillary column. The effects of gas-chromatography–mass-spectrometry (GC-MS) optimization conditions, headspace equilibrium temperature, headspace equilibrium time and standard solution preparation method on the determination of ethanol content in oil were discussed, and optimized measures were proposed. The experimental results showed that the measurement can be more accurate under the headspace temperature of 80 °C and the headspace time of 40 min, and relative standard deviation percentage (RSD%) could reach to 4.62% under this condition. It was also pointed out that, for the preparation of standard solution, the method which controlled the sampling volume of anhydrous ethanol by microliter syringe could make the peak area of ethanol chromatogram have a better linear relationship with the standard curve. Under the similar linear range, the goodness of fitting curve without diluting process could be as high as 0.9993, while the method of preparing the stock solution and diluting stepwise to obtain the fitting curve only had a goodness of 0.9910. The method was validated by standard addition recovery test, and the recovery values obtained were between 90.3% and 95.8%. The optimized method is of great significance for the measurement of ethanol dissolved in insulating oil.

## 1. Introduction

As the core equipment of the power system, the transformer is related to the safety and stability of the power system and needs to be monitored to ensure safe operation. Cellulose is widely used in transformers as the main solid insulation because of its good mechanical properties and electrical properties, and is widely found in nature [1]. In ordinary kraft paper commonly used in transformers, the content of cellulose is as high as 90% [2]. The aging process of insulating paper is irreversible, and replacing insulating paper is unrealistic. Therefore, it is generally believed that the aging state of the insulating paper is the key factor determining the remaining life of the oil-immersed transformer [3]. Cellulose is a natural polymer composed of glucose units connected by 1,4-β-glycosidic bonds [2]. The aging process of insulating paper is the process of breaking the macromolecular chain of cellulose, causing a decrease in the degree of polymerization (DP) of insulating paper [4]. The DP is an index to measure the size of a polymer molecule based on the number of repeating units, and is the average number of repeating units contained on the polymer macromolecular chain [5]. By directly measuring the DP of the insulating paper, the aging information can be clearly obtained [6,7]. However, measuring DP requires cutting off the power of the transformer in operation and may cause damage to the internal structure of the transformer [8]. Ethanol, a type of low molecular weight alcohol, has been discovered by scholars in recent years, which can be produced by breaking the chemical bonds of cellulose molecules and ethanol was proposed as a new marker for evaluating the insulation condition of oil-immersed transformers [2]. It has been found that the marker of ethanol has good stability in the operating temperature range of the transformer, and the presence of ethanol can be detected in transformers of different types of insulating paper [9,10,11]. Ethanol in oil can reflect the existence of local hotspots when the insulating paper is unevenly heated, while other traditional markers in oil can only reflect the overall insulation aging, which is the most potential advantage of ethanol markers [12,13]. Insulating oil samples were extracted from 21 field operation transformers, and the ethanol concentration was found to be about 5–640 μg/kg [14]. The detection methods at present of low-molecular-weight alcohols in insulating oil mainly include high-performance liquid chromatography (HPLC), gas chromatography (GC) [14,15], gas-chromatography–mass-spectrometry (GC-MS), spectrophotometry [16,17] and headspace-gas chromatography-mass spectrometry (HS-GC-MS) [1,15]. Spectrophotometry is an easy method to operate and takes less time, but the reproducibility and accuracy is not superior enough [18]. High-performance liquid chromatography and gas chromatography require tedious preparatory work, which takes a long time [18]. Gas chromatography equipped with mass spectrometry is a relatively standard method for the detection of alcohols and it is accurate for substance detection. Nonetheless, direct injection will cause more impurities, which inevitably contaminate the inlet, column and detector [19]. In 1960, the method of headspace analysis was first proposed [20]. In recent years, static headspace sampler has become an excellent tool for handling volatile organic compounds [21,22]. Many interferences can be effectively avoided if the headspace sampling technique is applied to the substance measurement and then combined by gas chromatography and mass spectrometry. The method has the advantage of sensitivity, accuracy and precision, reducing the amount of impurities entering the chromatographic system, and effectively reducing equipment contamination. The lower detection limit is a key advantage, and it can be detected even if the content of ethanol dissolved in the oil is only 4 μg/kg [19].

**Figure 1:**
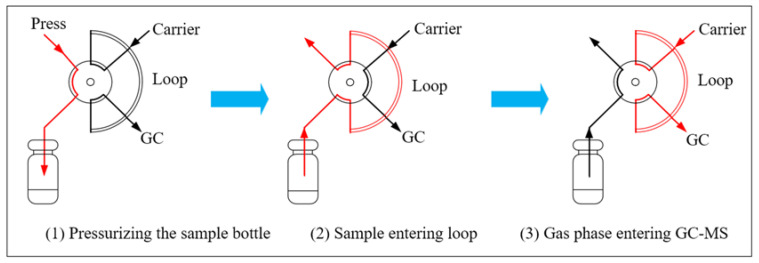
Sample loop working mode.

The headspace sampling technique is to put the sample into a closed container, in which samples are heated at a certain temperature for a period of time to balance the gas phase and the liquid phase, and extract the gas phase into the GC for analysis [22,23]. The headspace sampling technique is used for the pretreatment of volatile compounds. The sample vial is pressurized with the carrier gas first, then the gas of the sample, which achieves equilibrium, is filled with a sample loop by means of a six-way valve, and the gas passes through the transmission channel and enters the GC unit. The injection volume can be controlled by the sample loop which has strong heat resistance, and the sample loop avoids the absorption of compounds in the tube wall, greatly refrains sample loss, and ensures excellent reproducibility [24]. The injection principle [25] is shown in Figure 1.

However, in the detection and quantification process, alcohols are faced with the problem of insufficient accuracy. Sometimes, it is found in the actual experiment that the content may not be detected, or the quantitative result cannot reach the ideal condition.

In this study, the optimized method for the determination of ethanol content in transformer insulating oil by headspace–gas-chromatography–mass-spectrometry was proposed. The effects of GC-MS parameters, headspace equilibrium temperature and headspace equilibrium time on the peak area of ethanol in insulating oil were investigated. To mitigate the impact of the volatility and water absorption of ethanol, controlling the sampling volume of pure ethanol when preparing standard solutions is proposed. The optimized method has important practical significance for the detection of low-molecular alcohols and volatile organic compounds.

## 2. Materials and Instruments

### 2.1. Materials

Ethanol with a purity >99.9% and 25# mineral naphthenic transformer oil were applied to prepare the calibration samples. The insulating oil should be filtered through a 0.2 μm nylon membrane in advance to make it cleaner. Weidmann T4 insulating paper with 0.5 mm thickness and 25# mineral naphthenic insulating oil together constitute an insulating system for accelerated thermal aging experiments under laboratory conditions. The main characteristics of blank insulating oil and anhydrous ethanol are summarized in Table 1.

### 2.2. Instruments

An HSS86.50 Plus static headspace sampler (DANI, Cologno, Italy), which has a 1 mL sample loop, and GCMS-QP2020 (SHIMADZU, Tokyo, Japan), which includes a gas chromatograph and a mass spectrometer were applied to this experiment to conduct qualitative and quantitative analysis. A XPE204S high-precision electronic balance (METTLER TOLEDO, Zurich, Switzerland) was used to ascertain the mass of material and the precision of the electronic balance used is 0.01 mg. Twenty-milliliter headspace vials with a polytetrafluoroethylene (PTFE) sealing gasket were used to load samples for measurement.

The chromatography separation was performed by VF-624ms capillary column (Agilent, Santa Clara, CA, USA) of 60 m length, 0.25 mm diameter and 1.40 μm film thickness. The inlet temperature of mass spectrometer was maintained at 250 °C and mass-to-charge ratio (*m*/*z*) was 20–100 amu scanned in total ion count mode (TIC). Helium was adopted as a carrier gas and the split flow mode was adopted with a split ratio of 30:1. External standard calibration method was conducted to quantify the ethanol concentration based on integrated peak area.

## 3. Experimental Principle and Process Description

### 3.1. Selection of GC-MS Parameters

The key parameters of GC-MS that affect chromatographic separation include injection volume, split ratio, carrier gas flow rate, and column temperature [16]. In the actual detection process, abnormal phenomena such as the overlapping, trailing, and disappearing of chromatographic peaks may sometimes be encountered. By optimizing these parameters, better peak shape and separation effectiveness can be obtained, and the occurrence of abnormal peak shape can be avoided, which can make the qualitative analysis and quantitative analysis more accurate.

The capacity of column on the injection volume must be limited. If the injection volume is at a low level, some low-content components may be lost during transmission and cannot be successfully detected in GC-MS. Conversely, the column may be overloaded, resulting in excessive peak shape and peak overlap, reducing the resolution of chromatographic analysis. The split ratio indirectly affects the injection volume, the amount of sample entering the column decreases as the split ratio increases, and the response value can be improved by appropriately reducing the split ratio. Increasing the carrier gas flow rate will lead to an increase in the peak appearance time and make analysis speed faster, but this is unfavorable for the separation of the material being detected. It may be difficult to obtain a good peak shape of the measured material under the condition of a low-carrier gas flow, which will cause adverse effects in the analysis. Contrary to the constant temperature, the programmed temperature rise will be conducive to shortening the retention time of the high-boiling organic matter and facilitate material separation. The use of programmed temperatures also facilitates the removal of impurities in the final stage to eliminate residue in capillary column.

Reference [16] compared the effects of different injection volumes, split ratios, and carrier gas flow rates on the chromatographic peaks and set reasonable GC-MS parameters. Reference [1] and [2] used programmed temperature to separate low-molecular alcohols and other organics and obtain favorable results.

### 3.2. Selection of Equilibrium Temperature

The equilibrium temperature determines the pressure caused by the volatilization of organic matter in the sample vial and also affects the partition coefficient between the liquid phase and the gas phase [25]. Raising the heated temperature of the sample is one way to improve the sensitivity of headspace technique [26]. As the temperature increases, the amount of ethanol evaporated from the insulting oil increases, which plays an advantageous role in detection. However, the increase in temperature causes other high-boiling substances in oil to improve the possibility of volatilization, which will interfere with the result in some degree. In addition, excessive pressure may cause gas to escape when the syringe inserting into the sample vial, causing the gaseous sample to leak. Even the vial has the risk of exploding under high pressure and temperature, posing a safety threat to operator.

The boiling point of ethanol is about 78.5 °C. During the actual detection process, when the equilibrium temperature of the headspace sampler is at 60 °C, the ethanol in the insulating oil can be detected, indicating that the ethanol has shown a volatile phenomenon at this temperature. When the equilibrium temperature is above 100 °C, sometimes the detected ethanol content deviates significantly from the expected value. This may be because the high temperature causes the vial to be excessively pressurized, resulting in gas leaks when the syringe is inserted into the vial. The ethanol peak area was performed at temperature of 60, 70, 80, 90, and 100 °C, respectively. For the measurement, the equilibrium time of each sample to be detected was controlled to 60 min to keep samples in equilibrium adequately. At different temperatures, 10 mL standard solution in identical concentration of 7.9 mg/L was placed in a 20 mL headspace vial and tested 10 replicate samples to obtain the peak area at the corresponding temperature, and the relative standard deviation percentage (RSD%) was calculated soon afterwards. The relative standard deviation, also known as the variation coefficient, reflects the precision of the analysis results in the test work [27]. Therefore, by comparing the precision at each temperature, the optimal equilibrium temperature will be obtained.

### 3.3. Selection of Equilibrium Time

The required time for equilibrium of the sample in the headspace vial is essentially dependent on the diffusion rate of the sample composition molecules from the matrix to the gas phase [25]. Choosing a reasonable equilibrium time can assure the reproducibility of analysis and assure that the analysis is performed at equilibrium condition [23]. Increasing the temperature will increase the rate of diffusion, and the time to reach equilibrium will be shortened in the case of a fast diffusion rate. Too long an equilibrium time may cause problems, such as leak in the vial inlet, causing sample loss. After obtaining the optimal headspace equilibrium temperature by Section 3.1 and Section 3.2, then use 5, 10, 15, 20, 25, 30, 35, 40, 45, 50, 55 and 60 min as an equilibrium time, and observe the change rule of ethanol peak area in insulating oil. When the ethanol peak area no longer changed significantly, the optimal headspace equilibrium time will be obtained.

### 3.4. Standard Solution Preparation Method

For the quantitative methods of ethanol in insulating oil, there are mainly the internal standard method [1] and external standard method [2]. The external standard method does not need to purchase the internal standard materials, and only needs to prepare a standard solution for different concentration gradients of the substances being tested, which takes less time and is a relatively simple and economical quantitative method. Conventional standard solution preparation uses a step-by-step dilution method, in which anhydrous ethanol is added into the insulating oil, and then the solution will be diluted stepwise from a high concentration stock solution to a lower concentration. Under the influence of outside temperature, the volume of the stock solution will change due to the mechanism of heat expansion and cold contraction, so the concentration will change with the change in solution volume, and the concentration of the solution may have deviated from the theoretical value after dilution. In addition, the original solution may not be evenly mixed during the dilution process.

**Figure 2:**
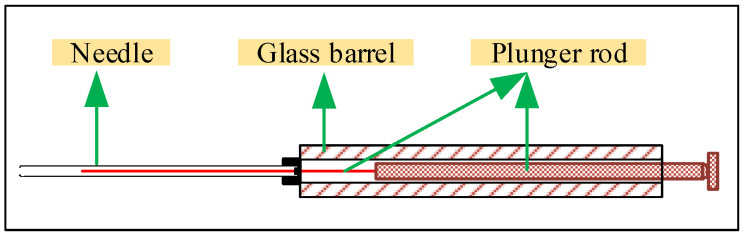
Structure of microliter syringe.

The sample volume can be controlled more precisely using a microliter syringe. The microliter syringe is available in a variety of sizes, 0.5, 1, 2, 5, 10, 20 μL, etc. The 5 μL volume and below microliter syringe can ensure that no liquid or gas remains in the syringe when the push rod reaches the 0-scale line. The microliter syringe needle reduces the adhesion of the liquid to the inner wall, which ensures an accurate sampling volume. The structure of the microliter syringe is shown in Figure 2 [22].

Dilution process requires an electronic balance with high precision and a high cost. It takes a longer time to prepare the stock solution, and the mass of the ethanol is inevitably lost to some extent because of its volatility. In addition, anhydrous ethanol, itself needed in stock solution, has a very low water content and will absorb moisture from the surrounding environment [28].

In order to investigate the evaporation rate of ethanol, two 100 mL volumetric flasks were taken at room temperature of 25 °C, containing 20 mL of insulating oil. The volumetric flask was placed in a tray of electronic balance, using a 5 μL microliter syringe to add ethanol and observe the number change of electronic balance. One group directly dropped ethanol above the level of the insulating oil, and one group dropped the ethanol below the level of the insulating oil. When the two numbers of balance were both 3.6 mg, the number change over time was recorded.

With the aid of a microliter syringe, the following two methods were used to prepare standard solution, and fitting curves were established for comparison. The concentration gradients of the two methods were controlled to be the same; the solutions of different concentrations need to be prepared three times, and the peak areas of the samples corresponding to each solution concentration were averaged.

#### 3.4.1. Method by Stepwise Dilution

The temperature was kept constant at 20 °C, and 1.000 g of ethanol was accurately added into a 1000 mL volumetric flask using an electronic balance. In order to prevent the rapid evaporation of ethanol, the volumetric flask should be added with a little insulating oil in advance. The total mass of the volumetric flask should be within the effective weighing range of the balance after adding the insulating oil. After adding ethanol, the insulating oil was continuously added to the volumetric flask, and the stock solution was set to a concentration of 1.000 g/L. In order to ensure that the ethanol is sufficiently evenly distributed in the insulating oil, the volumetric flask containing the stock solution should be shaken for 4 h in a constant-temperature water bath oscillator at a temperature of 20 °C. After the shock was completed, the stock solution was sealed and stored at 20 °C. Use a microliter syringe to extract the stock solution and dilute it by adding insulating oil to prepare ethanol standard solution concentrations of 0, 0.080, 0.160, 0.240, 0.320, 0.400, and 0.480 mg/L. The prepared standard solution should be stored in a 100 mL volumetric flask at 20 °C.

#### 3.4.2. Method by Controlling Ethanol Volume

When using a microliter syringe to extract liquid, the volume can be effectively controlled. The density of ethanol is known at a certain temperature. As long as the corresponding temperature is ensured, the mass can be calculated, thereby eliminating the step of weighing. It avoids the exposure of anhydrous ethanol to the air for a long time, which reduces the degree of volatilization and water absorption. Before using the microliter syringe to inject ethanol, a little insulating oil should be added into the volumetric flask in advance. Finally, add the insulating oil to the mark line with a plastic dropper. The ethanol was directly injected below the level of the insulating oil and then the needle of the microliter syringe should be shaken immediately. When the ethanol at the tip of the needle entered the insulating oil, the volumetric flask should be shaken again to make the solution mix well. Keeping the room temperature constant at 20 °C and using a microliter syringe, accurately extract 0, 0.1, 0.2, 0.3, 0.4, 0.5, and 0.6 μL of ethanol into a 1000 mL volumetric flask. According to the density of the purchased ethanol at 20 °C is 0.790 g/mL, the standard solution concentration calculated is 0, 0.079, 0.158, 0.237, 0.316, 0.395, 0.474 mg/L, respectively. The same as in Section 3.4.1, in order to make the ethanol mixed in the oil evenly, the constant temperature water bath oscillator is employed by shaking at 20 °C for 4 h to fully dissolve the ethanol in the oil. This process does not require the preparation of stock solution.

### 3.5. Methodology Validation

#### 3.5.1. Standard Addition Recovery Test

Standard addition recovery test is a commonly used method in chemical analysis. The accuracy and reliability of the results can be determined by measuring the recovery value. Take 100 mL of ethanol-free blank insulating oil and a 100 mL sample containing 0.1 mg/L ethanol, and use 10, 15, and 20 mg/L higher concentration ethanol solutions for standard addition. The addition volume is strictly controlled at 1 mL to reduce the change in sample volume. After the solution is thoroughly mixed evenly, take 10 mL and put it into a headspace vial. Repeated detection was performed three times on every sample before and after spiking under the same headspace conditions and gas chromatography mass spectrometry conditions, and then the recovery rate was calculated.

#### 3.5.2. Determination of Samples under Thermal Ageing Experiment

In order to speed up the aging process of insulating paper, scholars use heat-aging experiments at higher temperatures in the laboratory to simulate the aging of transformers. First, the 0.5 mm insulating paper and 25# naphthenic insulating oil were dried in a vacuum drying oven at 90 °C for 24 h to remove moisture. The dried insulating oil was put into an ultrasonic oscillator for 20 min to perform degassing operation. Then, take four 100 mL glass bottles and number them with A, B, C, and D, respectively. The oil-paper mass ratio of A, B, and D is 10:1 (oil 50 g, paper 5 g) while the ratio adopted by C is 15:1 (50 g oil, 3.33 g paper). Finally, the glass bottle was sealed, and the four oil–paper samples were subjected to accelerated heat aging at different temperatures. The insulating oil was extracted at different times, and the ethanol content was detected using the HS-GC-MS method. The two standard curves obtained in Section 3.4 were used to quantify the ethanol concentration in the oil. The main information of the aging experiment is shown in Table 2.

## 4. Results and Discussion

### 4.1. Optimal GC-MS Parameters

**Figure 3:**
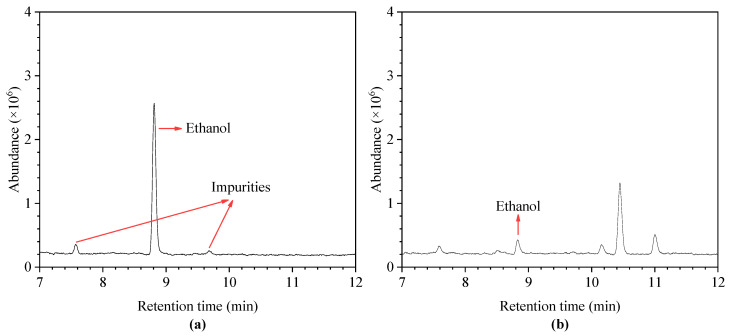
Chromatographic response of ethanol in insulating oil. (**a**) mixed solution of absolute ethanol and blank insulating oil, (**b**) insulating oil specimen extracted from a field transformer.

According to the discussion of GC-MS parameters in Section 3.1, referring to the parameter settings mentioned in the literature, gas chromatography detection of the insulating oil samples with ethanol was performed. A total of two samples were detected, one was a mixed solution of absolute ethanol and blank insulating oil (7.9 mg/L), and the other was an insulating oil specimen extracted from a field transformer (40 MVA) which has been in service for 15 years. The retention time of ethanol in the chromatogram was about 8.825 min, which had a good peak shape and good separation with other organic matter in insulting oil. This shows that the parameter setting of GC-MS meets the detection requirements. The main GC-MS parameters are shown in Table 3. The chromatographic response of ethanol in the standard sample and transformer oil sample is shown in Figure 3.

### 4.2. Optimal Equilibrium Temperature

**Figure 4:**
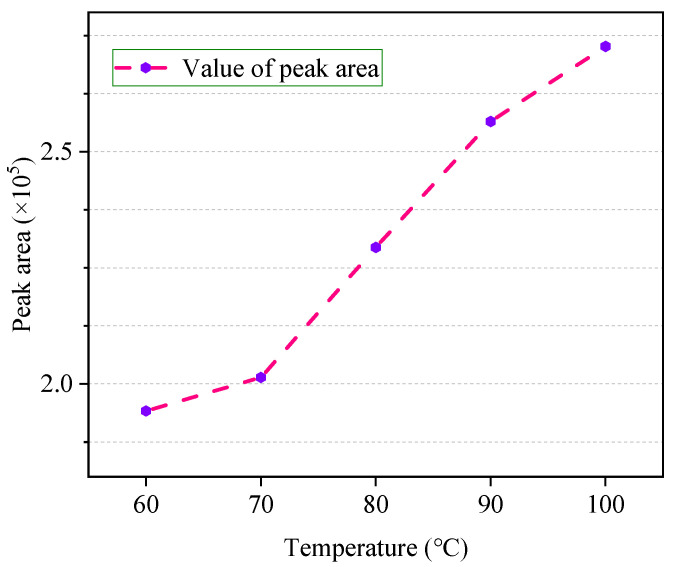
The change in ethanol peak area with headspace equilibrium temperature.

The relationship between the equilibrium temperature and the peak area is shown in Figure 4. It can be found that the equilibrium temperature is closely related to the peak area of ethanol. In the range from 60 to 100 °C, as the temperature increases, the peak area of ethanol gradually increases. Among them, the peak area has the fastest change rate from 70 to 90 °C. In the temperature range from 90 to 100 °C, as the temperature increases, the upward trend of ethanol peak area slows down, but the trend of peak area will continue to rise.

**Figure 5:**
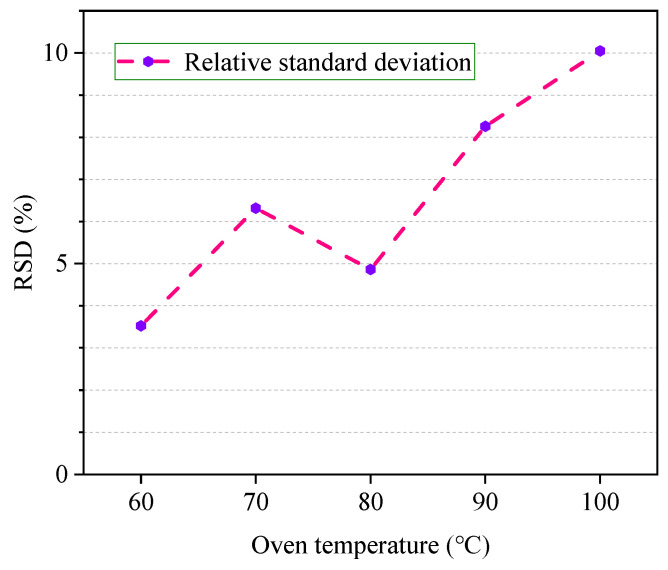
RSD% of ethanol peak area under different headspace temperatures.

The RSD% at different temperatures is shown in Figure 5. It can be concluded that at 60 °C, the RSD% has the lowest value, and with the increase in temperature, the RSD% has a significant rise at 70 °C. At 80 °C, the RSD% decreases again, and there is a trend toward higher temperatures, above 100 °C.

Although RSD% is the lowest at 60 °C, there may be a possibility that an undetectable problem may occur due to less content of ethanol in the gas phase, which may easily lead to erroneous judgment. Therefore, compared with 60 °C, the temperature of 80 °C ensures higher reliability and at this temperature, the RSD% reached 4.62%. Therefore, 80 °C is taken as the optimum equilibrium temperature.

### 4.3. Optimal Equilibrium Time

**Figure 6:**
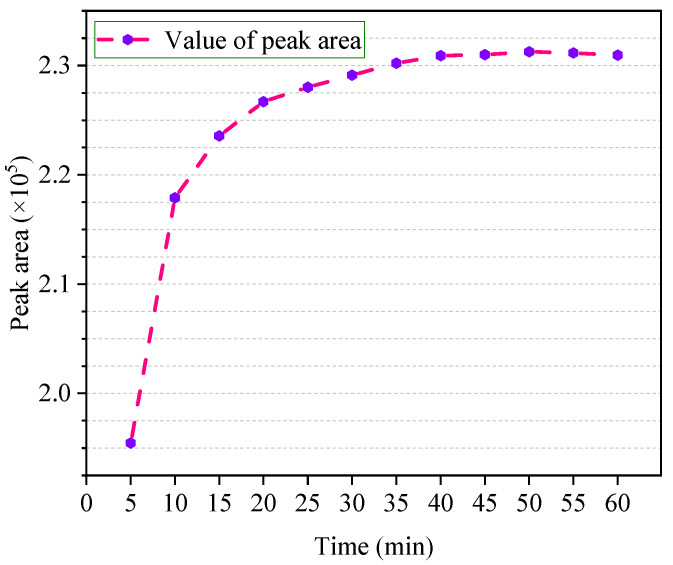
The change in ethanol peak area with headspace equilibrium time.

It can be seen from the Figure 6 that the peak area of ethanol is significantly lower than other temperatures at 5 min, indicating that there is still a large gap to achieving equilibrium conditions. The curve between 10 and 40 °C is a rising trend, but the trend rate gradually decreases. This means that, in a short period of time, the two-phase equilibrium cannot be achieved. As time goes by, the gas–liquid two phases gradually approach the equilibration, which also shows that if the equilibrium time is too short, it is unfavorable for detection. At 40 min, the curve no longer shows an upward trend. It can be considered that, at 40 min, the ethanol standard solution reached a two-phase equilibrium of gas phase and liquid phase and was stable. After 40 min, the curve showed a slight downward trend, which may have caused air leakage owing to the loose connection between the vial and the cap. Therefore, 40 min is taken as the optimum equilibrium time.

Based on the above analysis, the optimized headspace conditions are summarized in Table 4.

### 4.4. Comparison of Standard Solution Preparation Methods

**Figure 7:**
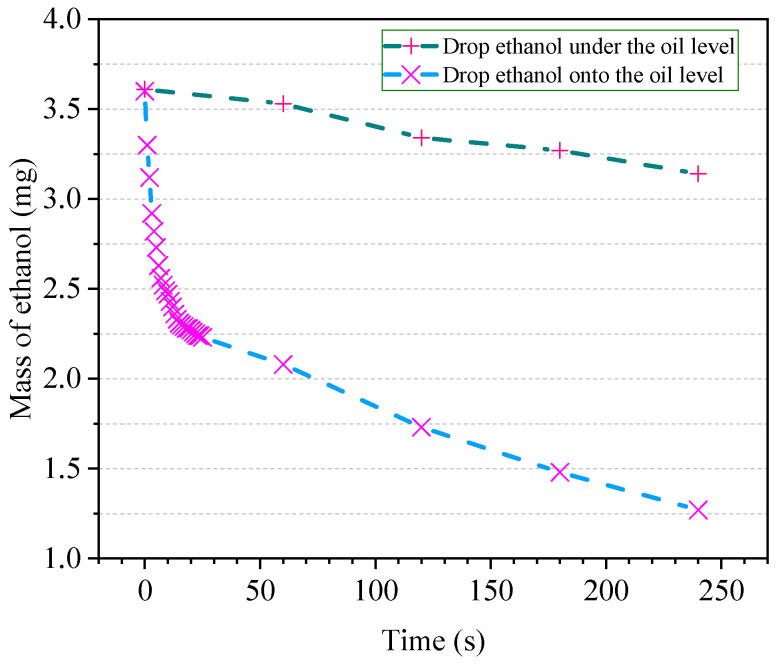
Evaporation condition of ethanol (25 °C).

It has been observed from Figure 7 that, when formulating the stock solution, if the ethanol is added directly to the volumetric flask, the number of the electronic balance will be gradually decreased before the addition of the insulating oil, indicating that the ethanol is in volatilization. If a small amount of insulating oil is added to the volumetric flask before adding ethanol, ethanol will not dissolve in the insulating oil at the beginning. Since the density of ethanol is lower than that of insulating oil, it will float on the oil surface, and ethanol will evaporate into the air. Tested at room temperature of 25 °C, the evaporation of ethanol is still very rapid; if the operation takes a long time, it will have a great impact on the actual concentration of the solution. Excessive working temperature will aggravate this phenomenon. Although improving the experimental environment, such as reducing the temperature, can reduce the loss of ethanol, this will undoubtedly increase the cost, and the construction of this environment is difficult.

If the insulating oil is added to the volumetric flask in advance and the ethanol is added below the level of the insulating oil, this situation will be significantly improved. As can be seen from Figure 7, shortening the time taken to prepare the solution is beneficial to reducing the evaporation loss of ethanol. Figure 7 compares the difference between dropping ethanol above the level of the insulating oil and below the liquid level. For the measurement of volatile matter, it is critical to ensure the reduction in volatilization, not only for the detection of ethanol, but also for the measurement of volatile organic compounds such as methanol. It is necessary to improve the measurement method and decrease the ambient temperature as much as possible.

**Figure 8:**
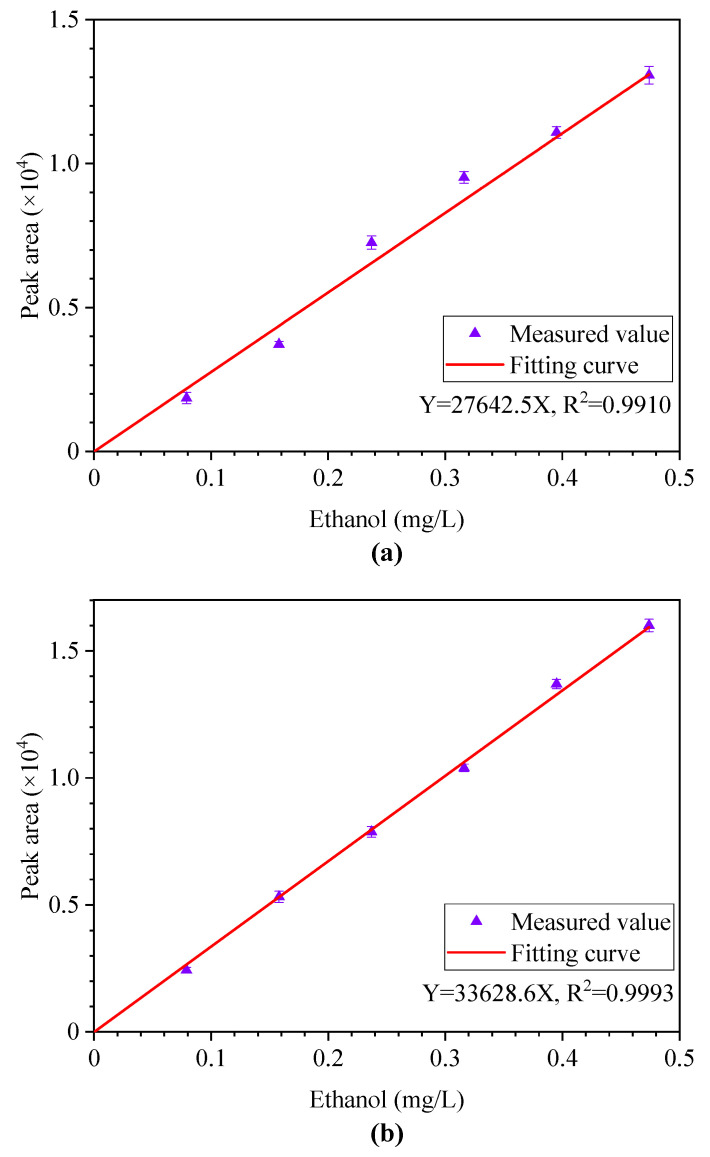
Standard curve obtained by two methods. (**a**) stepwise dilution method, (**b**) controlling the volume of ethanol method.

It should be noted that in this study, the following fitting curves were subjected to force coordinate origin (0, 0) crossing. Figure 8a reflects the standard curve for a stepwise dilution where Y = 27642.5X, R^2^ = 0.9910, and the linear range is from 0 to 0.480 mg/L. When the method of controlling the sampling volume of ethanol is adopted, the whole process will not take much time. The electronic balance is used to detect the preparation process, and it is found that the number of the electronic balance has hardly changed, which indicated that the amount of ethanol loss was very weak in a short time. Figure 8b reflects the standard curve without dilution, where Y = 33628.6X, R^2^ = 0.9993, and the linear range is from 0 to 0.474 mg/L.

By comparison, the standard curve obtained by the undiluted method has a preferable linear relationship, and the peak area deviation is lower compared to the dilution method. The reason for this phenomenon may be that the rapid preparation process reduces the evaporation loss and water absorption of anhydrous ethanol. Another possible reason for this is that the solution before dilution is not sufficiently well mixed when the stepwise dilution method is applied. Therefore, for the preparation of standard solution with different concentration gradients, it is very necessary to reduce the time of dilution, and the dilution must be carried out on the premise that the origin liquid has been mixed evenly.

### 4.5. Results of Methodology Validation

**Figure 9:**
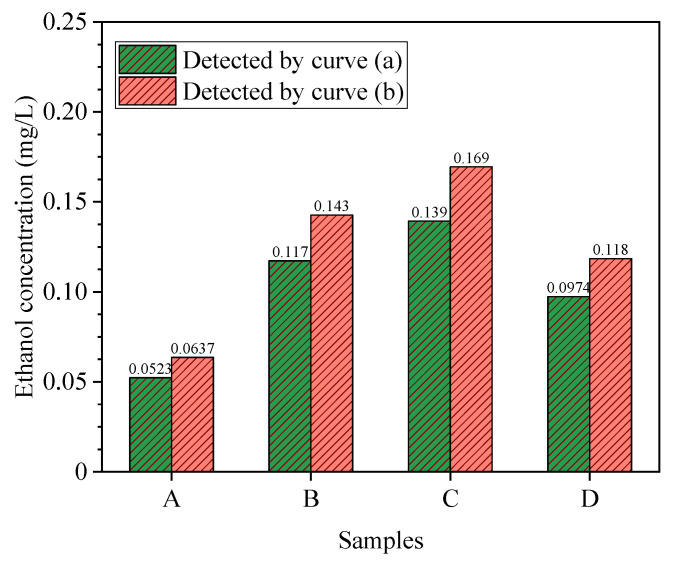
Comparison of standard curves between (a) and (b) applied in ethanol quantification in the samples under laboratory conditions.

It can be known from Table 5 that the average recovery rate is between 90.3% and 95.8%. This shows that the method has higher accuracy in actual measurement. The measurement results of different samples after aging in the laboratory are shown in Figure 9, in which curve (a) represents the standard curve obtained under stepwise dilution method, and curve (b) represents the standard curve obtained by directly controlling the volume of anhydrous ethanol. By comparing the ethanol content in the insulating oil samples of A, B, C, and D with different aging times, it is found that the ethanol concentration measured by standard curve (a) drawn by stepwise dilution is lower. The concentration of the four samples exactly meets the linear range of the standard curve, but this does not mean that the above standard curve is also satisfactory for the measurement of ethanol content in other samples. If it does not meet the requirements, a stepwise dilution to prepare the standard solution may be necessary.

## 5. Conclusions

It is unrealistic to directly sample the insulating paper of the transformer to evaluate the insulation state of the transformer. However, it is convenient to detect the ethanol of aging indicator by sampling the insulating oil of transformer. In this paper, the influence of headspace equilibrium temperature and equilibrium time on the measurement results of ethanol content in insulating oil was studied and the differences between two standard solution preparation methods were compared. The method was verified by recovery test and transformer insulation oil sample detection. The above method also has a certain degree of significance for the measurement of other volatile organic compounds by the method of headspace–gas-chromatography–mass-spectrometry. Several points to summarize this study are shown below:

(1) The chromatographic peak area of ethanol in the sample was significantly affected by the headspace temperature and equilibrium time. In a certain range, the peak area increases gradually with the extension of the above two factors;
(2) The reproducibility of the experiment results is best at about 80 °C and gas–liquid two phases equilibration will be obtained at about 40 min. This means that the ethanol detection results obtained under this condition are more scientific;
(3) The standard curve obtained by controlling the sample volume of anhydrous ethanol when preparing standard solution can have a better linear relationship compared with the dilution method. Under the premise of satisfying the concentration range needed in detection, it is better to avoid by stepwise dilution when manufacturing the standard solution. If the standard curve cannot meet the detection requirements, a stock solution should be prepared and diluted stepwise.

## Figures and Tables

**Table 1:** Main characteristics of blank insulating oil and anhydrous ethanol.

Properties	Insulating Oil	Ethanol
Density (20 °C), kg/m^3^	895.7	-
H2O, ppm	29	-
Density (20 °C), g/mL	-	0.790
H2O, %	-	0.01

**Table 2:** The main information of the samples.

Number	Aging Time (Days)	Temperature (°C)	Oil-Paper Ratio
A	10	100	10:1
B	5	110	10:1
C	5	130	15:1
D	3	150	10:1

**Table 3:** Main instrument parameters of gas-chromatography–mass-spectrometry (GC-MS).

Instrument Parameters	GC	MS
Split ratio	30:01:00	-
Helium carrier gas flow rate	1.04 mL/min	-
Interface temperature	250 °C	-
Pressure	52.6 kPa	-
Temperature program	40 °C for 10 min, then 10 °C/min to 210 °C, hold on 20 min.	-
Ion source	-	Electron impact
Ionization energy	-	70 eV
Acquisition mode	-	Scan
mass-to-charge ratios	-	20–100 amu
Ion source temperature	-	200 °C
Quadrupole temperature	-	150 °C

**Table 4:** Instrument parameters of Headspace.

Instrument Parameters	Headspace Sample
Oven temperature	80 °C
Equilibrium time	40 min
Loop temperature	110 °C
Transfer line temperature	120 °C
Vial pressure	15 psi
Pressurization time	0.15 min
Injection time	0.50 min
Loop fill time	0.15 min

**Table 5:** Recovery test results.

Initial Sample Concentration (mg/L)	Standard Addition Concentration (mg/L)	Ethanol Content after Standard Addition (μg)			Average Recovery (%)
0	10	9.5	8.7	8.9	90.3
0	15	14.4	12.8	13.7	90.9
0	20	18.6	18.5	19.4	94.1
0.1	10	19.6	18.9	19.4	93.0
0.1	15	24.3	24.3	24.5	95.8
0.1	20	27.8	28.3	28.6	91.2
